# Barriers to Receiving Proton-Craniospinal Irradiation for Pediatric Medulloblastoma Patients at a Rural Tertiary Care Center

**DOI:** 10.7759/cureus.102293

**Published:** 2026-01-26

**Authors:** Melisa Pasli, Megan Goins, Michael C Larkins, George Edwards, Dayana Gonzalez, Cathleen Cook, Andrew W Ju, Aidan Burke

**Affiliations:** 1 Medicine, East Carolina University (ECU) Brody School of Medicine (BSOM), Greenville, USA; 2 Radiation Oncology, East Carolina University (ECU) Brody School of Medicine (BSOM), Greenville, USA; 3 Emergency Medicine, Boonshoft School of Medicine, Wright State University, Dayton, USA; 4 Internal Medicine, East Carolina University (ECU) Brody School of Medicine (BSOM), Greenville, USA; 5 Pediatrics, East Carolina University (ECU) Brody School of Medicine (BSOM), Greenville, USA

**Keywords:** barriers to care, craniospinal irradiation (csi), pediatric medulloblastoma, proton beam therapy, treatment barriers

## Abstract

Introduction: Medulloblastoma is predominantly a childhood cancer, with craniospinal irradiation (CSI) being a common treatment modality. Here, we discuss barriers to proton-CSI for rural pediatric medulloblastoma patients.

Methods: Pediatric patients <25 years old with a diagnosis of medulloblastoma were identified from a tumor registry at a rural tertiary academic center. A chart review was conducted to identify specific barriers to proton CSI, namely distance and time to the nearest proton therapy facility, median household income, and race. Descriptive and statistical analyses using the Kruskal-Wallis H test and chi-square were conducted in IBM SPSS Statistics for Windows, Version 28 (Released 2021; IBM Corp., Armonk, New York, United States) to describe this cohort and their barriers.

Results: Of the 18 pediatric medulloblastoma patients identified, no significant associations were found between barriers of interest and discussion or pursuit of proton-CSI. Median follow-up time was five years. All three patients who received proton-CSI were Caucasian patients. Non-Caucasian patients had shorter median travel times, with the association approaching significance (p = 0.068). No significant associations were found between county, parent employment and marital status, tumor classification, risk stratification, and the reception of proton-CSI or its discussion.

Conclusion: To the best of our knowledge, this is the first study on barriers to accessing proton-CSI for pediatric medulloblastoma patients without in-state proton-CSI centers. Our study provides insight into geographic, social, and financial barriers, allowing clinical teams to identify solutions to overcome these barriers. Advocacy for access to care on behalf of this vulnerable patient population will expand treatment access on a state and national level.

## Introduction

Medulloblastoma is the most common pediatric brain tumor malignancy, representing approximately 20% of all brain tumors found in children and 40% of all posterior fossa tumors [1,2]. Notably, 80% of patients diagnosed with medulloblastoma are under the age of 15 [3].

Treatment of medulloblastoma is multi-modal, including surgery, chemotherapy, and, more recently, craniospinal irradiation (CSI) [4,5]. It should be noted, though, that chemotherapy was noted to be added as an accepted treatment only more recently, around 2000 [6]. CSI has become a staple of medulloblastoma therapy over the past several decades, since 30-40% of patients have CSF spread at the time of diagnosis [2]. CSI has been shown to prevent spinal relapse and is the first curative therapy for medulloblastoma. In 1953, Edith Paterson and R.F. Farr from Christie Hospital and the Holt Radium Institute in Manchester, England, conducted the first widely regarded study demonstrating the efficacy of irradiating the whole central nervous system for treatment of medulloblastoma; in their study, five of seven patients survived at least five years, with the longest surviving over 10 years [7] A little over a decade later, a landmark Toronto study showed the majority of patients survived more than five years using CSI [5].

There are two primary forms of CSI: photon-CSI and proton-CSI. The original form of CSI, photon-CSI, poses several notable risks to medulloblastoma patients. One major adverse effect is organ toxicity, including ototoxicity, cardiotoxicity, endocrine and neurocognitive dysfunction, and growth impairment [3]. These photon-CSI toxicities have been linked with reduced functionality and quality of life in the long-term. Another major adverse effect is CSI-induced secondary malignancy. Retrospective studies of patients treated with photon-CSI revealed a 9.3-19% incidence of secondary malignancy by age 30 [8]. A large meta-analysis showed that childhood cancer survivors have up to over 50 times the risk of developing a subsequent CNS neoplasms, with nearly all of these survivors having exposure to CSI, sometimes in a dose-dependent fashion [9]. Further, the Childhood Cancer Survivor Study has shown increasing mortality rates due to secondary malignancy. The death rate from secondary malignancy surpasses that of all other causes 25 years post-diagnosis [8].

Since 2012, the standard of care for CSI in higher-income countries has evolved to incorporate proton beam therapy [4,5]. Introduced in 1946 by Robert Wilson, its main advantage over photon-CSI is significantly reduced off-target irradiation. This is due to the Bragg Peak phenomenon, whereby the proton beam concentrates high doses of radiation to the tumor field, while delivering reduced levels of radiation to adjacent tissue [3]. A meta-analysis of studies comparing photon and proton-CSI found that compared to photon-CSI, proton-CSI had a better dose distribution, spared more out-of-field organs, caused less normal organ dysfunction, and caused less secondary malignancy. Due to these advantages, a nearly 230-case shift from photon-CSI to proton-CSI was observed between 2010 and 2012 among pediatric patients treated for medulloblastoma in the United States (that is to say, 230 cases that would have been photon-CSI were changed to proton-CSI).

Despite these advantages, proton-CSI centers are few in number in the United States, at 46 in total [10]. This is in the context of linear accelerators and given the size of the United States. Consequently, access to these centers is a prominent barrier to care. As centers are only available in certain regions, a burden exists for families outside the vicinity of a nearby proton-CSI center. This leads to families relocating to a new location for weeks to receive adequate treatment [11].

Proton-CSI center infrastructure, equipment, and assembly are expensive, estimated to range from $25 million to $200 million, and require a plethora of resources and trained staff to ensure successful operation. Care coordination between oncologists, institutions, and primary care physicians, facilitation of temporary residence for patients during treatment, and the requirement of insurance authorization may cause treatment delay for patients. There is also a risk of proton centers failing (financially), as was seen in Indiana in 2014 [12]. These factors are particularly pronounced for patients living far from an established proton-CSI center [11].

In addition to the cost of construction, there is the cost of operation to consider. The total cost of proton-CSI for pediatric patients has been estimated at approximately $80,000 as of 2013 (roughly $110,000 in 2025), and has been associated with approximately 3.46 additional quality-adjusted life years (QALYs) as compared to traditional photon radiotherapy [13].

Here, we conducted a retrospective study identifying barriers to receiving proton-CSI for medulloblastoma patients in a rural tertiary care center approximately 135 miles from the nearest proton therapy center. The primary objective of this study is to assess if there is an association between geographic and socioeconomic factors and ultimate receipt of proton-CSI among pediatric patients lacking an in-state facility that offers such treatment, compared to those that ultimately receive such treatment despite it being offered.

## Materials and methods

Overview

We conducted an IRB-approved retrospective study (UMCIRB 22-002292) using patients diagnosed with medulloblastoma from a pediatric tumor registry at a tertiary care center in a rural geographic region of eastern North Carolina. Of note, this tertiary care center offered radiotherapy for the majority of patients in our study, but did not offer proton therapy. The closest center (assuming availability) is approximately 135 miles away.

Eligibility

Eligible patients were diagnosed with medulloblastoma at our institution between 2000 and 2022 and were aged <25 at the time of diagnosis.

Data collection

All eligible patients were characterized demographically by sex, race, age at diagnosis, half-decade corresponding to their date of diagnosis, distance from our institution in miles and minutes, county, median household income as defined by county of residence, sibling status, parent marital status, parent employment status, and insurance approval barriers. Distance and time from our institution were derived using Google Maps (Google LLC, Mountain View, CA, USA) for car travel in March 2023. Since our study includes patients from our local catchment area in a rural area of eastern North Carolina, patients receiving radiation therapy farther from our institution were more likely to have resources for extensive travel. For this reason, we selected distance to our institution as a reasonable metric to assess travel barriers to care. Median household income was calculated using the United States Census Bureau registration for each patient’s county zip code, using 2021 dollars, from the years 2017-2021 (United States Census Bureau, 2022). The barriers of interest we identified included having transportation to our institution, being able to afford travel expenses, having parents who were able to travel, family care logistics, inpatient and outpatient medical needs, and delays in therapy. Independence was designated by patients being self-dependent in terms of not relying on outside support for the variables. Length of follow-up was calculated using each patient’s date of diagnosis and the date of their last follow-up visit at our institution. We also specified patients participating in clinical trials.

Patient medulloblastomas were staged using Modified Chang Staging and stratified into standard versus high risk [14]. Medulloblastomas were also classified by histologic type, genetic mutations, histopathologic findings, and presence of tumor markers.

Patient treatment regimens and outcomes were described in terms of whether they received proton-CSI, CSI radiation dose, total radiotherapy dose, number of tumor resections, and development of secondary malignancy and recurrence. The cut-off date for survival status was July 21, 2023. Our geographic barriers of interest were distance and estimated travel time to our institution. Our financial barrier of interest was the median household income based on the county of residence. Our demographic barrier of interest was a racial breakdown of Caucasian vs. non-Caucasian (composed of Black and Hispanic patients).

Statistical analyses

Barriers to care were compared between patients who received proton-CSI and those who did not, as well as between patients who had documented discussions about proton-CSI with their provider and those who did not. Due to our sample size and distribution, nonparametric tests were used for statistical calculations to assess associations and differences between the sub-groups outlined above. Dichotomous variables were compared to outcomes such as reception of radiation and discussion of proton therapy using Fisher’s exact test. Continuous variables, such as time and distance to our institution, were compared using Kruskal-Wallis or chi-squared testing. All statistical analyses were conducted in IBM SPSS Statistics for Windows, Version 28 (Released 2021; IBM Corp., Armonk, New York, United States) with an alpha level of 0.05.

## Results

Of 18 total patients, three (17%) received proton-CSI, and 15 (83%) did not. Of these 18 patients, 11 (61%) had documented discussions about proton-CSI in their medical records, while seven (39%) did not. Our patient population consisted of 13 (72%) Caucasian and five (28%) non-Caucasian patients. Sex breakdown was 11 (61%) male and seven (39%) female.

Table 1 demonstrates the demographic distribution for our study population based on the overall characteristics of each patient. The distance between each patient’s address and our institution ranged from 2.1 miles (six minutes) to 180.1 miles (169 minutes), with a median of 64.8 miles (76 minutes). Calculated distance and estimated travel time were strongly correlated (Pearson’s r = 0.999). Median household income by county ranged from $37,832 to $76,345, with a median of $52,124 and a mean of $51,966. All of our sub-groups, consisting of patients that received proton therapy and those that did not, and those that had the discussion about proton therapy and those that did not, were shown to have the same median household income. Ten (56%) patients had parents who were still married, while eight (44%) patients had parents who were divorced or separated. Eight (44%) patients had parents who were both employed, one (6%) had both parents unemployed, six (33%) had one employed, and one unemployed parent, two (11%) were independent, and one (6%) had an unknown parent employment status. Fifteen (83%) patients had siblings, while three (17%) did not.

**Table 1:** Demographic Breakdown of Study Population A table displaying information on the patients identified in this study, including demographic, income, and insurance information. * M: mother; F: father SSI: Supplemental Security Income

Patient	Age (Years)	Race	Sex	Median Household Income	SSI	Insurance	Parents’ Marital Status	Parents' Employment Status*	Sibling
1	10	Caucasian	Male	$52,124	No	TRICARE PRIME/HUMANA MILITARY	Married	F: employed, M: unemployed	Yes
2	12	Caucasian	Male	$41,798	No	BCBS MEDICAID-HEALTHY BLUE	Married	F: unemployed, M: employed	Yes
3	22	Caucasian	Female	$56,325	No	BCBS	Married	Independent	Yes
4	15	Caucasian	Male	$54,732	No	MEDICAID	Married	Both employed	Yes
5	8	Caucasian	Male	$37,832	No	AMERIHEALTH MCAID ADV	Married	F: employed, M: unemployed	Yes
6	25	Caucasian	Female	$61,805	No	BCBS MEDICAID-HEALTHY BLUE	Divorced	Independent	No
7	7	Black	Female	$54,732	Yes	AMERIHEALTH MCAID ADV	Divorced	Both unemployed	Yes
Patient	Age (Months)	Race	Sex	Median Household Income	SSI	Insurance	Parents’ Marital Status	Parents' Employment Status*	Sibling
8	8	Black	Female	$41,974	Yes	AMERIHEALTH MCAID ADV	Married	Both employed	Yes
9	20	Black	Female	$50,422	No	CIGNA	Divorced	Both employed	Yes
10	4	Caucasian	Male	$50,422	No	BCBS	Divorced	F: employed, M: unemployed	Yes
11	1	Caucasian	Male	$61,805	No	TRICARE/HUMANA MILITARY	Divorced	Both employed	No
12	7	Caucasian	Male	$76,345	Yes	CIGNA	Divorced	Both employed	Yes
13	1	Caucasian	Male	$54,732	No	TRICARE	Married	F: employed, M: unemployed	No
14	17	Caucasian	Male	$41,974	No	BCBS	Married	Both employed	Yes
15	3	Caucasian	Male	$52,124	No	BCBS	Married	Both employed	Yes
16	0.9	Black	Male	$45,744	No	MEDICAID	Divorced	Both employed	Yes
17	13	Hispanic	Female	$45,766	No	MEDICAID	Married	Not available	Yes
18	2	Caucasian	Female	$54,732	No	MEDICARE	Divorced	F: employed, M: unemployed	Yes

Table 2 represents the medulloblastoma characteristics regarding each patient’s tumor classification, histopathological characteristics, tumor markers, genetic mutations, and risk stratification. The medulloblastoma tumor classifications were histologically refined using the WHO Classification of Tumors of the Central Nervous System [15], which yielded seven (39%) classic medulloblastoma cases, three (16.7%) desmoplastic/nodular, three (16.7%) anaplastic, one (5.6%) medulloblastoma with extensive nodularity (MBEN), and four (22%) that were not otherwise specified (NOS). The patients were graded by risk using Modified Chang Staging and Risk Stratification, based on their T and M staging [14,16,17]. A total of 16 (89%) patients had standard risk, while two (11%) had high risk.

**Table 2:** Medulloblastoma Characteristics of Study Population A table displaying information regarding the histology, risk stratification, stage, and CSF status of the patients identified with medulloblastoma as a part of this study. CSF: cerebrospinal fluid; NOS: not otherwise specified; MBEN: medulloblastoma with extensive nodularity

Patient	Diagnosis	Half-Decade of Diagnosis	Histologic Type	Mutations, Tumor Markers, and Histopathology	CSF Status	T Stage	M Stage	Risk Stratification
1	Medulloblastoma	2015-2020	Classic	MYCN gain, heterozygous MTHFR mutation C677T,Neurofilament:neg., NSE:pos., MIB-1/Ki67 index:variable	Negative	T3a	M0	Standard
2	Medulloblastoma	2020-2025	Mixed Large Cell/Anaplastic	Isochromosome 17q, MYC amplification, 1q gain, Neurofilament:pos., NSE:pos., CD56:pos., CD34:neg., EMA:neg.	Negative	T3a	M0	Standard
3	Medulloblastoma	2020-2025	Classic	GFAP:pos., Neurofilament: pos., NSE:patchy w/ <5%, MIB-1/Ki67 index:40-50% maximally, CD56:pos., CD3:neg., CD20:neg.	Negative	T2	M0	Standard
4	Medulloblastoma	2015-2020	NOS	GFAP:patchy, Neu-N:pos., MIB-1/Ki67 index: 50%, INI-1:pos., EMA:neg.	Negative	T1	M0	Standard
Patient	Diagnosis	Half-Decade of Diagnosis	Histologic Type	Mutations, Tumor Markers, and Histopathology	CSF Status	T Stage	M Stage	Risk Stratification
5	Medulloblastoma	2020-2025	Anaplastic/Large Cell	Neurofilament:neg., MIB-1/Ki67 index:increased, CD56:pos., CD34:neg., S100:pos.	Positive	T3a	M3	High
6	Medulloblastoma	2020-2025	Classic	TP53 wild-type	Negative	NOS	M0	Standard
7	Medulloblastoma	2020-2025	Classic	Loss of chromosome 3/gain of chromosome 7/subclinical gain of chromosome 10, Neurofilament:neg., NSE:pos., MIB-1/Ki67 index:~20% overall, CD56:pos., INI-1:pos.	Negative	T3a	M0	Standard
8	Medulloblastoma	2015-2020	Desmoplastic/Nodular	C-MYC amplification, 1p36 deletion	Negative	T3a	M0	Standard
9	Medulloblastoma	2015-2020	Classic	Chromosome 6 monosomy, YAP1:pos.	Negative	T2	M0	Standard
10	Medulloblastoma	2000-2005	NOS	Not available	Negative	T3a	M0	Standard
11	Medulloblastoma	2010-2015	NOS	GFAP:neg., Neurofilament:pos., MIB-1/Ki67 index:variable, BAF-47:pos., EMA:neg.	Negative	NOS	M0	Standard
Patient	Diagnosis	Half-Decade of Diagnosis	Histologic Type	Mutations, Tumor Markers, and Histopathology	CSF Status	T Stage	M Stage	Risk Stratification
12	Medulloblastoma	2015-2020	Classic	Isochromosome 17q	Negative	T3a	M0	Standard
13	Medulloblastoma	2000-2005	MBEN favored (Diff. Dx=Desmoplastic)	Vimentin:pos., NCAM:pos.	Positive	NOS	M3	High
14	Medulloblastoma	2010-2015	Anaplastic	GFAP:pos., NSE:pos., MIB-1/Ki67 index:>80% focally, CD56:pos., Chromogranin:patchy	Negative	T3a	M0	Standard
15	Medulloblastoma	2015-2020	Classic (with focal severe anaplasia)	GFAP:pos., Vimentin:pos., NSE in >70%, MIB-1/Ki67 index:variable, CD99:pos., AE1/AE3 cytokeratin:dot-like	Negative	T3a	M0	Standard
16	Medulloblastoma	2000-2005	Nodular/Desmoplastic	GFAP:pos., Neurofilament:neg.	Negative	NOS	M0	Standard
17	Medulloblastoma	2005-2010	NOS	GFAP:neg.	Negative	T3a	M0	Standard
18	Medulloblastoma	2000-2005	Desmoplastic	GFAP:pos., Neurofilament:neg., NSE:pos.	Negative	T2	M0	Standard

Table 3 details the receipt of radiation and proton-CSI, radiotherapy specifications, participation in clinical trials, overall survival outcome, length of follow-up, cancer recurrence, number of tumor resections, and any secondary malignancies. While all 18 patients received radiation therapy, only three (17%) patients received proton therapy. Six patients (33%) received 3D-conformal radiation therapy (CRT) since they were diagnosed prior to 2017, when our institution used traditional photon therapy techniques. Eleven (61%) patients received intensity-modulated radiation therapy (IMRT) or volumetric modulated arc therapy (VMAT) since they were diagnosed after 2017, when our institution switched to more modern photo therapy techniques. One patient received an unknown radiation technique, as they were treated at an outside facility without available records. Three (17%) patients participated in clinical trials. We had 16 (89%) of 18 patients still alive as of July 21, 2023. Fourteen (78%) of the patients in our study required only one tumor resection, while four (22%) patients required two. Even though only two (11%) patients experienced cancer recurrence, a total of two (11%) had secondary malignancies, which is on par with secondary malignancy rates observed in retrospective studies as referenced above. Patient 10 developed a papillary thyroid cancer 17 years after treatment, and patient 18, with Gorlin syndrome, developed multiple basal cell carcinomas after eight years of treatment and a right schwannoma after 14 years of treatment. Median follow-up was five years after treatment.

**Table 3:** Treatment Characteristics and Outcomes of Study Population A table displaying the treatment information, follow-up length, and outcomes for patients identified with medulloblastoma. CSI: craniospinal irradiation; IMRT: intensity-modulated radiation therapy; VMAT: volumetric modulated arc therapy; 3D CRT: three-dimensional conformal radiation therapy; RT: radiation therapy; XRT: external beam radiation therapy; COG: Children’s Oncology Group; ACNS: Atypical Teratoid/Rhabdoid Tumor and Medulloblastoma Clinical Trial Series; UNC: University of North Carolina; ECU: East Carolina University

Patient	Proton Therapy	Radiation Therapy	Radiation Specifications	Clinical Trial Participant	Deceased	Recurrence	Secondary Malignancy	Length of Follow-Up (Months)
1	Yes	Yes	According to SJMB12 St. Jude (IMRT/VMAT)	No	No	No	No	59
2	Yes	Yes	Protons St. Jude 36 Gy CSI, 18 Gy boost, per ACNS 0332 (IMRT/VMAT)	Yes	No	No	No	17
3	Yes	Yes	Transfer to Northwestern for protons and egg harvest (IMRT/VMAT)	No	No	No	No	1
4	No	Yes	23.4 CSI, 30.6 boost at UNC per ACNS0331 reg A (3D CRT)	No	No	No	No	68
5	No	Yes	39.6 Gy CSI, 16.2 boost per ACNS0332 (IMRT/VMAT)	Yes	No	No	No	9
6	No	Yes	23.4 CSI, 30.6 boost ECU Health (3D CRT)	No	No	No	No	10
7	No	Yes	36 Gy, 19.8 Gy, per ACNS0332 at ECU Health ()	Yes	No	No	No	22
8	No	Yes	23.4 Gy CSI, 30.6 Gy boost at ECU Health (IMRT/VMAT)	No	No	No	No	61
9	No	Yes	30.6 CSI, 23.4 Gy boost brain at ECU Health (IMRT/VMAT)	No	No	No	No	71
10	No	Yes	23.4 CSI, 32.4 Gy boost at ECU Health (3D CRT)	No	No	No	Yes (papillary thyroid after 17 years)	269
11	No	Yes	XRT at Duke Sept 2011 (technique unknown)	No	No	Yes	No	45
12	No	Yes	23.4 CSI, 30.6 boost ECU Health (3D CRT)	No	No	No	No	85
13	No	Yes	St. Jude protocol, chemo at first, then chemo and RT (3D CRT)	No	Yes	Yes	No	13
14	No	Yes	23.4 CSI, 30.6 boost ECU Health (3D CRT)	No	No	No	No	114
15	No	Yes	23.4 CSI, 30.6 boost ECU Health (3D CRT)	No	No	No	No	80
16	No	Yes	As per COG P9934, 32.4 Gy CSI, 18 Gy boost (3D CRT)	No	No	No	No	188
17	No	Yes	Treatment 2007, ECU Health 30.6 Gy, 23.4 Gy boost, 3D plan (3D CRT)	No	Yes	No	No	Unknown
18	No	Yes	23.4 CSI, 32.4 Gy boost at ECU Health (3D CRT)	No	No	No	Yes (multiple basal cell carcinomas eight years after treatment, schwannoma 14 years after treatment, patient with Gorlin syndrome)	Unknown

Table 4 identifies specific barriers of interest regarding discussion of proton therapy, insurance approval, patient travel, and therapy logistics. Eleven (61%) patients had a discussion with their provider about proton therapy, four (22%) experienced insurance approval barriers, three (17%) could not afford travel, three (17%) had lack of transportation, one (6%) had parents unable to travel with them, four (22%) had family care conflicts, four (22%) had inpatient medical needs, five (28%) had outpatient medical needs, and three (17%) had a delay of therapy.

**Table 4:** Barriers to Proton-CSI for Study Population A table displaying identified potential barriers to patients receiving craniospinal irradiation (CSI) for treatment of medulloblastoma was identified through this study.

Patient	Discussed Proton Therapy	Insurance Approval Barrier	Unable to Afford Travel	Distance to Our Institution (Miles)	Time From Our Institution (Mins)	Lack of Transportation	Parent Unable to Travel With Child	Family Care Logistical Conflicts	Inpatient Medical Needs	Outpatient Medical Needs	Delay in Therapy
1	Yes	No	No	57.9	75	No	No	No	No	No	Yes
2	Yes	Yes	No	26.9	33	No	No	Yes	No	Yes	No
3	Yes	Yes	Yes	43.1	58	Yes	No	No	No	Yes	No
4	Yes	No	No	96.7	115	No	No	No	Yes	Yes	No
5	Yes	No	No	83.4	82	Yes	No	No	Yes	Yes	Yes
6	Yes	Yes	No	84.9	103	No	No	Yes	No	No	No
7	Yes	Yes	Yes	71.8	82	Yes	No	Yes	Yes	Yes	No
8	Yes	No	Yes	21.5	26	No	Yes	Yes	No	No	No
9	Yes	No	No	2.1	6	No	No	No	No	No	No
10	Yes	No	No	10.4	14	No	No	No	No	No	Yes
11	Yes	No	No	74	89	No	No	No	Yes	No	No
12	No	No	No	180.1	169	No	No	No	No	No	No
13	No	No	No	72.1	85	No	No	No	No	No	No
14	No	No	No	26.6	33	No	No	No	No	No	No
15	No	No	No	56	72	No	No	No	No	No	No
16	No	No	No	69.8	77	No	No	No	No	No	No
17	No	No	No	19.9	25	No	No	No	No	No	No
18	No	No	No	81.6	95	No	No	No	No	No	No

Tables 5-6 demonstrate how sex, race, estimated travel distance, median household income based on county, parent employment and marital status, tumor classification, and risk stratification varied between the patients who received proton-CSI or had a discussion on such matters, and those who did not. No significant associations were found between these variables and the reception of proton-CSI or its discussion.

**Table 5:** Selected Barriers to Proton-CSI based on Pursuit of Therapy Analysis of selected barriers to patients receiving radiotherapy for medulloblastoma using Fisher's exact and Kruskal-Wallis testing. CSI: craniospinal irradiation

Sub-group of Interest	Sex	Race	Median Distance From Our Institution	Median Household Income	Parent Marital Status	Parent Employment Status	Tumor Classification	Risk Stratification
Received proton-CSI (n = 3)	2 Males; 1 Female	3 Caucasian; 0 Non- Caucasian	71.8 miles; 82 minutes	$52,124	3 Married; 0 Divorced/Separated	0 Both employed; 3 Other	2 Classic; 1 Non-Classic	3 Standard; 0 High
Did not receive proton-CSI (n = 15)	9 Males; 6 Females	10 Caucasian; 5 Non- Caucasian	57.9 miles; 68 minutes	$52,124	7 Married; 8 Divorced/Separated	8 Both Employed; 7 Other	5 Classic; 10 Non-Classic	13 Standard; 2 High
Significance of Association	Fisher’s p > 0.99	Fisher’s p = 0.52	Kruskal-Wallis p = 0.44 (H = 2.31)	Kruskal- Wallis p = 0.81 (H = 1.42)	Fisher’s p = 0.22	Fisher’s p = 0.22	Fisher’s p = 0.53	Fisher’s p > 0.99

**Table 6:** Selected Barriers to Proton-CSI based on Provider Discussion of Therapy Analysis of medulloblastoma patients' treatment with radiotherapy based on the discussion of treatment with proton therapy. CSI: craniospinal irradiation

Sub-group of Interest	Sex	Race	Median Distance From Our Institution	Median Household Income	Parent Marital Status	Parent Employment Status	Tumor Classification	Risk Stratification
Discussed with provider (n = 11)	6 Males; 5 Females	8 Caucasian; 3 Non- Caucasian	59.8 miles; 75 minutes	$52,124	6 Married; 5 Divorced/Separated	4 Both Employed; 7 Other	5 Classic; 6 Non-Classic	10 Standard; 1 High
Did not discuss with provider (n = 7)	5 Males; 2 Females	5 Caucasian; 2 Non- Caucasian	69.8 miles; 77 minutes	$52,124	4 Married; 3 Divorced/Separated	4 Both Employed; 3 Other	2 Classic; 5 Non-Classic	6 Standard; 1 High
Significance of Association	Fisher’s p = 0.64	Fisher’s p > 0.99	Kruskal-Wallis p = 0.75 (H = 2.57)	Kruskal-Wallis p = 0.96 (H = 0.51)	Fisher’s p > 0.99	Fisher’s p = 0.63	Fisher’s p = 0.64	Fisher’s p > 0.99

Compared to the patients who received proton-CSI, those who did not receive proton-CSI had, on average, a longer median travel distance by 20.76 miles (16.2 minutes), though this finding was not significant. All three patients who received proton-CSI were Caucasian, had parents who were still married but not both employed, and a standard risk. The group that did not receive proton-CSI was composed of 10 (67%) Caucasian patients and five (33%) non-Caucasian patients, with eight (53%) having parents divorced or separated and seven (47%) married, and 13 (87%) standard risk, with two (13%) high risk. Neither of our two high-risk patients received proton-CSI.

Compared to the patients who did not discuss proton-CSI with their providers, those who had discussions had, on average, a shorter median travel distance by 20.24 miles (17.34 minutes). Also, there were eight (73%) Caucasian patients and three (27%) non-Caucasian patients who discussed proton-CSI with their provider, while five (71%) Caucasian patients and two (29%) non-Caucasian patients did not.

Table 7 shows our racial breakdown across the following variables: median travel distance from our institution, median household income based on county of residence, parent marital and employment status, tumor classification, and risk stratification. We found that Caucasian patients had a longer travel distance (by almost threefold), higher median county household incomes, and a higher rate of married parents. The only two patients with high-risk disease were both Caucasian.

**Table 7:** Selected Barriers to Proton-CSI based on Race Analysis of barriers to craniospinal irradiation (CSI) for medulloblastoma based on patient race, divided into Caucasian vs. Non-Caucasian.

Sub-group of Interest	Median Distance From Our Institution	Median Household Income	Parent Marital Status	Parent Employment Status	Tumor Classification	Risk Stratification
Caucasian (n=13)	72.1 miles; 85 minutes	$54,732	8 Married; 5 Divorced/Separated	5 Both Employed; 8 Other	5 Classic; 8 Non-Classic	11 Standard; 2 High
Non-Caucasian (n=5)	21.5 miles; 26 minutes	$45,766	2 Married; 3 Divorced/Separated	3 Both Employed; 2 Other	2 Classic; 3 Non-Classic	5 Standard; 0 High
Significance of Association	Kruskal-Wallis p = 0.07 (H = 4.13)	Kruskal-Wallis p = 0.20 (H = 2.47)	Fisher’s p = 0.61	Fisher’s p = 0.61	Fisher’s p > 0.99	Fisher’s p > 0.99

## Discussion

Treatment access for pediatric patients with medulloblastoma is an important problem that has not been well assessed. Pediatric cancer patients who reside in a rural setting often do not have access to advanced therapies, such as proton therapy, that are available to others. Many factors may play a role in this disparity, and although prior studies have focused on and analyzed pediatric patients receiving proton therapy at large institutions, this study puts proton therapy reception into perspective with a population that may not have adequate access to this preferred modality of treatment. The aim of this unique study was to encourage equitable treatment for this understudied patient population, with consideration of its needs and limitations to receive proton therapy. In our study, we considered several socioeconomic factors that may impact patients' ability to receive proton therapy and/or may prompt discussion with the provider of proton therapy for pediatric cancer patients living in rural counties surrounding our tertiary center. We considered factors such as sex, race, median distance from our institution, median household income, family marital status, employment status, tumor classification, and risk stratification to determine if associations exist. Thus far, to our knowledge, there are no studies with a focus on identifying barriers for proton-CSI reception in hospitals that do not have an in-state proton center and that serve a rural population.

Proton-CSI centers are capital-intensive to create and require a host of healthcare professionals to operate, restricting the number of centers in the United States. North Carolina does not possess any such centers, though one center is under construction as of 2023. This center will be approximately 230 miles from our institution, which will impose a significant financial and geographic burden on patients. The closest proton-CSI center for patients in this study was in a neighboring state, approximately 150 miles away, closer than the facility under construction. However, it is a privately operated center where, in general, insurance approval is required before consideration of treatment. Due to historic delays and uncertainties in obtaining insurance approvals across state lines within the crucial time frame from surgery to begin radiation therapy, we were more reluctant to refer to this center. While a viable option, additional logistical and bureaucratic barriers exist for out-of-state patients, including extreme difficulties with coordinating hospital transfer. Countries such as Canada and France have created national initiatives to expand access to and construction of proton therapy centers nationally, though the geographic distribution of the United States and the intersection between state and federal guidelines create additional nuance to this issue [18,19].

Given the cost mentioned regarding treatment with proton therapy, it is not a surprise that cost can be a prohibitive factor for patients obtaining treatment. Proton therapy costs around two to three times as much as traditional radiotherapy, with a significant restriction being the size and advanced technology required for proton treatment [20]. However, it has been noted that while the upfront cost of proton therapy is greater than that of traditional radiotherapy, such as IMRT, the cost of adverse events, such as hearing loss and growth hormone deficiency, outweighs the initial upfront cost in the long run (on a scale of decades) [21]. While not the focus of this analysis, consideration of the high upfront costs for proton therapy is important, and goes hand in hand with our suggestion for more case management support for patients later in this paper.

The literature is limited in identifying and analyzing social determinants of health in the context of pediatric cancer patients [22]. Although there have been studies assessing how socioeconomic status (SES), including marital status and employment variables, impacts pediatric cancer survival [23], few studies have been done to determine how SES affects access to proton therapy care for this vulnerable patient population. Shen et al. found that, in addition to race, other factors such as primary insurance type, level of neighborhood median income, and level of education determined access to proton therapy [24]. Though that study did not specifically consider marital and employment status, a connection between these SES factors should be considered to improve access to this therapy. Our study is novel in that we assessed marital and employment status variables as potential limiting factors to determine their impact on the reception of proton therapy in this patient population. Although Fischer's exact analysis demonstrated a non-significant association (p = 0.22), on chi-square analysis, we found that both employment status (p = 0.09) and marital status (p = 0.09) approached significance.

Pediatric patients are a particularly vulnerable population for accessing radiotherapy, as they are oftentimes completely dependent on a caregiver for transportation, scheduling, payment, and support. Disparities between cost and resources may be pronounced between urban and rural areas [25], and this disparity may greatly impact families in a rural setting with a child diagnosed with cancer. Such families reported an increased financial burden compared to families in an urban setting. Plausible explanations have been reported as decreased access to suitable healthcare and lower incomes [26]. In our study, there was no difference in median household income for those receiving protons versus those not receiving protons (p = 0.81). It should be noted, though, that the power of such an analysis is relatively poor, given that only three patients received proton therapy (of 18).

There have been studies on pediatric patients receiving proton therapy. Though proton therapy is considered an effective modality for the treatment of medulloblastomas in the pediatric population, race has been reported as an important factor when assessing access to proton radiotherapy, as Black pediatric patients may have reduced access and are less likely to receive such treatment. Bitterman et al. found that Black pediatric patients were less likely to receive proton therapy than their Caucasian counterparts, even when controlling for factors such as institution location and presence of metastasis [27]. Similarly, in our study, all the patients who received protons were Caucasian, although this association was not statistically significant (p = 0.24). Shen et al. reported that these racial disparities for pediatric patients may also be due to referral patterns [24]. Etminanighasrodashti et al. found that no association existed between race and travel distance to the radiotherapy provider [28]. Conversely, Rhodes et al. found that Black adult patients had a lower probability of traveling > 60 miles for cancer treatment compared to non-Black patients [29]. The demographic composition for the county of our home institution is approximately 52.7% Caucasian and 36.5% Black [30]. In our study, non-Caucasian patients exhibited overall shorter median travel times than Caucasian patients, with the association approaching significance (p = 0.068). This finding may be attributed to travel barriers for this population, which could indicate racial disparity for proton therapy access. The smaller representation of non-Caucasian pediatric patients in our study cohort and in those who received proton therapy may be an additional support for this theory.

The American Society of Clinical Oncology (ASCO) has emphasized the necessity of reevaluating access disparities for cancer care in which race may play a role [31]. This may be particularly important for proton therapy, as this modality yields the advantage of decreasing the development of secondary cancers. Proton-CSI is known to decrease the probability of developing such malignancies compared to photon-CSI. Sethi et al. modeled the 10-year cumulative incidence of radiation-induced secondary malignancy for pediatric patients receiving proton therapy, which was less than for those receiving photon therapy (0% vs. 14%, respectively; p = 0.015) [32]. Gondi et al. postulated that, due to the ethical consequences of randomizing children to receive treatment that delivers high doses of radiation, randomized studies are not done comparing protons to conventional X-ray therapy (photons) [33]. As such, nonrandomized trials are utilized to determine the benefit of proton therapy in overcoming radiation therapy-related side effects that may significantly impair quality of life while maintaining equal disease control [33,34]. In our study, none of the patients who received proton-CSI experienced recurrence or secondary malignancy, while three of the 15 patients who did not receive proton-CSI experienced recurrence and/or secondary malignancy. Median follow-up during our study was five years after treatment. This may be related to the length of follow-up and potentially highlights the necessity of proton therapy to prevent recurrence and secondary malignancies [35].

Several potential limitations of our study exist. First, this was a retrospective study relying mostly on reported information found within each patient’s chart, some of which can be subjective to the treating clinicians. Our retrospective study design is also limited by referral bias, whereby providers may selectively offer proton therapy based on perceived family logistical barriers. We also recognize that institutional protocols may have evolved over the 22-year period of our study. We faced challenges in identifying significant relationships in the data due to our small sample size. Comparing performance status before and after proton-CSI for patients with various barriers to care was a metric of interest that we could not evaluate due to the retrospective nature of our study. Future longitudinal prospective studies with larger sample sizes and greater clinical homogeneity could better uncover a relationship between medical barriers to proton therapy and the discussion and pursuit of proton therapy through the use of multivariate analysis. We propose that future studies further break down financial barriers to patients based on out-of-pocket costs, deductibles, and financial assistance using health economics models. Further elucidating the reasons for proton therapy referrals would also shed light on whether social factors play a role in a successful versus unsuccessful referral. Additionally, it should be noted that the distance from our institution to the patient's domicile is an indirect assessment of barriers to proton therapy specifically, and future work may benefit from analysis of this directly. Finally, our analysis was limited to identifying broad categories of barriers associated with treatment, and future studies would benefit from a more granular analysis. Our categorization of barriers to care could also be considered subjective, influencing the ultimate outcome of this analysis. It would also be preferred that detailed information on insurance approval and cost be available, though it was not a part of this analysis. Future efforts would be augmented by developing a cost-benefit analysis to determine the population necessary to support a proton therapy center.

Our study underscores the importance of analyzing various factors contributing to disparities of care in this understudied population, and improvement will require a coordinated care approach [36]. Solutions are warranted to overcome these barriers to receiving proton-CSI. Increasing the amount of in-state proton facilities would significantly ease distance barriers for patients, though it would admittedly be an expensive solution given the cost challenges described in the Introduction. A more cost-effective option would be expanding case manager services for coordination of appointments and connecting patients with local, state, and out-of-state resources. While our institution regularly uses case management to assist patients receiving radiotherapy, the burden on case managers is profound. This is exacerbated by the challenges our institution faces in serving rural patients due to the wide catchment area we serve. Implementing interdisciplinary teams for treatment has been shown to improve patient access to radiotherapy [37-39]. Reducing the disparities between urban and rural areas would require continuing reform, with the purpose of increasing access to healthcare in rural areas, encouraging recruitment, and improving training of rural providers [25].

## Conclusions

We conducted an exploratory analysis on barriers to proton-CSI for rural patients in a state without access to this treatment modality. We reported demographic and disease characteristics for 18 pediatric medulloblastoma patients at our institution. We also conducted statistical comparisons of geographic, financial, and racial barriers to care among patients who received and did not receive proton-CSI, as well as between those who discussed proton-CSI with their provider and those who did not. There is a trend toward patients having married parents being more likely to receive proton therapy and having both parents employed being less likely to receive proton therapy, though our study results are limited by a small sample size. As proton therapy has become part of the standard of care for medulloblastoma, delays or inability to access this treatment modality could have significant implications for the clinical outcomes of less fortunate patients. This study provides insight into barriers encountered by pediatric patients and their families and, therefore, may aid clinicians in mitigating these barriers. Overcoming these barriers to care may allow for optimal treatment and may reduce the risk of CSI-induced secondary malignancy and toxicity.
